# Knowledge graph and CBR-based approach for automated analysis of bridge operational accidents: Case representation and retrieval

**DOI:** 10.1371/journal.pone.0294130

**Published:** 2023-11-07

**Authors:** Hui Xu, Yuxi Wei, Yonggang Cai, Bin Xing

**Affiliations:** 1 School of Economics and Management, Chongqing University of Posts and Telecommunications, Chongqing, China; 2 Chongqing Innovation Center of Industrial Big-Data Co. Ltd., National Engineering Laboratory for Industrial Big-Data Application Technology, Chongqing, China; 3 College of Computer and Information Science, College of Software, Southwest University, Chongqing, China; Universiti Teknikal Malaysia Melaka Fakulti Teknologi Maklumat dan Komunikasi, MALAYSIA

## Abstract

Bridge operational accident analysis is a critical process in bridge operational risk management. It provides valuable knowledge support for responding to newly occurring accidents. However, there are three issues: (1) research specifically focused on the past bridge operational accidents is relatively scarce; (2) there is a lack of mature research findings regarding the bridge operational accidents knowledge representation; and (3) in similar case retrieval, while case-based reasoning (CBR) is a valuable approach, there are still some challenges and limitations associated with its usage. To tackle these problems, this research proposed an automated analysis approach for bridge operational accidents based on a knowledge graph and CBR. The approach includes case representation and case retrieval, leveraging advancements in computer science and artificial intelligence. In the proposed approach, the case representation involves the adoption of a knowledge graph to construct multi-dimensional networks. The knowledge graph captures the relationships between various factors and entities, allowing for a comprehensive representation of accidents domain knowledge. In the case retrieval, a multi-circle layer retrieval strategy was innovatively proposed to enhance retrieval efficiency. Three target cases were randomly selected to verify the validity of the proposed methodology. The combination of a knowledge graph and CBR can indeed provide useful tools for the automated analysis of bridge operational accidents. Additionally, the proposed methodology can serve as a reference for intelligent risk management in other types of infrastructures.

## Introduction

Bridge engineering indeed plays a crucial role in people’s lives. Over the past few years, there has been a significant increase in the construction of bridges worldwide, encompassing various types such as highway bridges, railway bridges, river crossing bridges, and sea crossing bridges, among others. Notably, China has been at the forefront of bridge construction, with a total number of modern bridges exceeding 1 million. Additionally, China boasts an impressive feat of having 90 out of the world’s top 100 high bridges that were newly built in the 21st century [1]. The operation of bridge is indeed vulnerable to various types of risks. Over the past 30 years, China has experienced more than 300 bridge operational accidents, encompassing accidents such as terrorist attack, blast threat, dangerous goods transportation, overload transportation, and bridge pier collision. Accident reports provide information to understand why and how events occur. Extracting information from these previous accidents, including accident types, causes, locations, and response measures, can provide invaluable knowledge for accident risk mitigation [2]. The hidden knowledge among past accidents, such as accident frequency, accident distribution, et al., can also be extracted [3]. By leveraging the lessons learned from past accidents, authorities and engineers can enhance bridge safety measures and reduce the likelihood of future accidents.

According to the nature of bridge operational accidents, the knowledge derived from the accidents exhibits distinct topological characteristics. To further enhance our understanding of accident information, there has been a significant focus on conducting topological analyses of bridge accident networks. Multi-dimensional networks, which encompass diverse types of nodes and edges, have the potential to offer more comprehensive information for topological analyses of bridge operational accidents. However, it is worth noting that existing studies have predominantly focused on single-dimensional complex networks. These networks typically consist of homogeneous nodes, such as cause-effect relationship edges [2]. Furthermore, the widely used topological indicators are primarily derived from single-dimensional networks, such as degree, betweenness centrality, and clustering coefficient [4]. Applying these indicators to reveal the inherent features of multi-dimensional networks with multiple types of nodes and edges becomes challenging. In this research, the use of a knowledge graph was considered to explore bridge operational accidents. The knowledge graph was proposed by Google in 2012 to improve the performance of search engines using semantic search information accumulated from various sources [5]. Knowledge graphs are powerful tools for representing entities, attributes, and relationships in a structured and interconnected manner [6]. In a knowledge graph, entities are represented as nodes, while relationships between entities are represented as edges or links. This structured representation forms the basic units of the graph, often referred to as "entity–relation–entity" triples [7]. In addition, knowledge graphs have the capability to automatically extract knowledge triples from both structured and unstructured data. By integrating, processing, and inferring information, they can form complex semantic networks of knowledge [5]. Therefore, a knowledge graph is indeed a database that connects various entities with attributes through relationships. Therefore, this study utilizes a knowledge graph modeling method to conduct analysis on bridge operational accidents. In addition, this research also incorporates a case-based reasoning (CBR) approach to extract risk response experience from past bridge operational accidents. CBR mimics human cognition and learning by adapting previously successful similar cases to generate appropriate solutions. CBR has gained increasing attention in emergency response management [8]. By leveraging the knowledge and experiences derived from historical cases, CBR can enhance decision-making processes and improve the effectiveness of emergency response in bridge operational accidents.

The purpose of this study is to build a knowledge graph of bridge operational accidents based on historical accident data, and further integrate CBR to conduct research on similar case retrieval. By identifying similar cases, the study aims to provide insights and references for resolving new operational accidents in bridge management through case experience reuse. The research framework is as follows. Section 2 is the literature review, which presents the research gaps. Section 3 provides an introduction to case representation and case retrieval methods, specifically focusing on the use of knowledge graphs and CBR. In Section 4, an application results of the methodology is presented, demonstrating its practical implementation in real bridge operational accidents. This application serves to validate the feasibility and efficacy of the proposed case representation and retrieval methods. Section 5 is the discussion. Finally, in Section 6, the paper concludes with a summary of the findings and presents the theoretical and practical contributions of this study.

## Literature review

As bridge is a crucial component of infrastructure engineering, many methods adopted in infrastructure operational risk research are also applicable to exploring operational risks in bridges. Analyzing past accident data is indeed a widely used method to understand operational risks in infrastructure. Elvik et al. adopted negative binomial regression models to identify factors that explain systematic variation in the number of injury based on 1368 bridge accidents in Norway from 2010 to 2016 [9]. Liu et al. proposed a network theory-based approach to understanding railway operational accidents. The topological analysis for the accidents provides a practical way to extract the accidents knowledge from accident reports [10]. Zhou and Lei explored the paths between latent and active errors for 407 railway accidents/incidents by using the Human Factors Analysis and Classification System [11]. Hughes et al. described a multi-lingual ontology to identify specific classes of railway safety incident based on 5065 safety incident reports [12]. Drawing on existing research findings, this study conducts the automated analysis of bridge operational accidents based on the collected bridge operational accidents. A knowledge graph has been adopted to establish the structure of accident cases and support CBR in extracting risk response experiences.

### Knowledge graph application and construction

The application of knowledge graphs in risk management research has proven to be valuable. Specifically, in the domain of gas system risk management, a temporal knowledge graph have been constructed and utilized for predicting the risk level of the system [13]. In the field of long-distance oil and gas pipeline emergency management, the establishment of a knowledge graph has been employed to develop an emergency task recommendation model [14]. For railway hazard management, a knowledge graph has been developed for hazard identification and risk assessment in railway operations [15]. In the field of construction risk management, a knowledge graph of accidents has been used for accident analysis and prevention [6]. Besides, a knowledge graph-based decision support approach was proposed for the operational hazard management of utility tunnels based on structured field investigation data and unstructured text files [5].

The construction of a knowledge graph typically involves the combination of multiple technologies and methods. Ontologies provide a generalized data model that captures shared properties and relationships among general types of things, and don’t include information about specific individuals within a particular domain. By utilizing ontologies as a framework, specific case information can be added to create a knowledge graph [16]. For example, Guo and Goh developed an ontology to facilitate knowledge sharing and reuse in the design of active fall protection systems (AFPS) [17]. Fang et al. integrated computer vision algorithms with ontology models to develop a knowledge graph in the field of hazard identification and safety regulations [18]. Combining with the BERT-BiLSTM-CRF algorithm, Wu et al. constructed an ontology model for knowledge representation of construction safety accidents [19]. Gan et al. established a knowledge graph for ship collision accidents by utilizing natural language processing (NLP) techniques based on 241 investigation accident reports [20]. Employing BiLSTM and Conditional Random Field (CRF) model, Chen et al. proposed a knowledge graph for critical infrastructure protection that aimed to address information gaps in management across different industries and timeframes [21]. Besides, deep learning techniques have also been integrated into the knowledge graph construction process. For instance, a graph-based deep learning framework was proposed to identify accident-injury type and body part factors [22]. In addition, a deep learning-based method was conducted to extract and represent relations in the form of knowledge graph-based queries that describe fall protection requirements, which helps decompose complex requirements into manageable units [23].

### CBR approach in risk management

Case-based reasoning (CBR) is an alternative, increasingly popular approach for designing expert systems [24]. The 4R model, consisting of case retrieval, reuse, revision, and retention, has been established [25]. CBR has been widely adopted in similar accident case retrieval studies and making risk response decisions. In the aspect of similar case retrieval, one example is the work by Lu et al. integrated various steps of construction safety risk management into a unified framework utilizing CBR, the k-Nearest Neighbor (KNN) algorithm and rough set theory [26]. Similarly, aiming to enhance the efficiency of retrieving similar cases, Goh and Guo developed an online knowledge-based system, FPSWizard, which adopts a combination of CBR and rule-based reasoning (RBR) [27]. In the aspect of making risk response decisions, Fan et al. proposed a pragmatic method for generating project risk response strategies based on CBR [28]. Chen et al. developed a framework for a decision-support system that utilizes CBR with a nearest-neighbor retrieval (NNR) search mechanism for adjudicating construction industry occupational accidents [29]. Liu et al. proposed a practical method for generating dispute settlement solutions for international construction projects using CBR [30]. Ayhan and Tokdemir proposed an innovative safety assessment methodology that utilizes Artificial Neural Network (ANN) and CBR to predict possible scenarios and determine preventative actions for safety incidents, and to estimate the outcomes of incidents specifically in terms of severity [31].

### Gaps in research

According to existing studies, the following three research gaps can be concluded:

1. The experience-oriented approach in infrastructure risk management emphasizes the importance of leveraging valuable experiences from similar historical cases to address new problems. This approach has been successfully applied in various risk management domains, such as road bridge risk management and railway operational accidents management. However, research specifically focused on applying this approach to bridge operational accident management is relatively scarce.
2. There is a lack of mature research findings regarding the bridge operational accidents knowledge representation. Indeed, knowledge graphs have found applications in risk management research across multiple domains, including gas system risk management, long-distance oil and gas pipeline emergency management, railway hazard management, construction risk management, utility tunnels’ hazard management, etc. These applications have demonstrated the effectiveness of knowledge graphs in representing accident cases. However, the application of knowledge graphs in bridge operational management, particularly for understanding bridge operational accidents, has been relatively limited.
3. The CBR approach is valuable in enriching reference cases for experience mining. However, there are still some challenges and limitations associated with its usage. Besides, it is common to combine CBR with other methods to leverage their respective strengths and enhance the overall performance. Some approaches that can be combined with CBR include: KNN, rough set theory, RBR, NNR, ANN, etc. Nevertheless, there has been limited research on the combined use of CBR and knowledge graphs, and it is an area worth exploring further.

This study aims to address these three gaps. It focuses on conducting an automated analysis of bridge operational accidents. A knowledge graph representing bridge operational risk knowledge is constructed. Besides, the CBR approach has been innovatively utilized and integrated with knowledge graphs.

## Methods

### Knowledge representation based on knowledge graph

To study bridge operational accidents, a specific knowledge graph called the Bridge Operational Accident Knowledge Graph (BOAKG) is constructed. The first step involves extracting knowledge entities from the accident cases, which represent concepts or entities in the real world. The second step is to identify the relationships among these knowledge entities. These relationships capture the semantic connections between the entities and reflect the dependencies and interactions within the accident scenarios. Finally, the knowledge entities and their relationships are mapped into a network graph, forming the BOAKG.

#### Extraction of knowledge entities and attributes

The knowledge entities in the BOAKG represent fundamental units of knowledge related to bridges. It is important to note that the extent of accident data coverage determines the range of corresponding knowledge entities [2]. To extract these entities, the complete contents of the accident cases are preserved and be the base of the text segmentation. In English text, spaces between words serve as natural delimiters, making it relatively easy to identify word boundaries. However, in Chinese text, the delimiters do not indicate word boundaries as clearly. Instead, they mark the boundaries between characters, sentences, and paragraphs, which poses a challenge when it comes to determining the boundaries between individual words. There are various Chinese text segmentation tools available, including THULAC, NLPIR, LTP, Jieba, HanLP, etc. LTP and HanLP are more focused on semantic analysis and provide additional functionalities beyond just text segmentation. These tools use advanced models and techniques to perform tasks such as named entity recognition and part-of-speech tagging. Jieba, THULAC, and NLPIR are primarily designed to accurately segment Chinese text into individual words or tokens. Jieba has several advantages that make it a popular Chinese text segmentation tool. One is its ability to accurately segment Chinese sentences and long phrases, while also precisely punctuating them [32]. It also offers several other functionalities, including keyword extraction and word position query [5]. Besides, Python is a popular programming language in Jieba application for natural language processing and data analysis due to its portability, readability, and ease of use [6]. Thus, the Jieba Chinese text segmentation algorithm is employed in this study. This algorithm follows three steps. Firstly, word graph scanning based on a prefix dictionary. The algorithm scans the text to identify possible words using a dictionary that contains word prefixes. Secondly, finding all the possibilities of Chinese characters in sentences. Thirdly, finding the most probable combination based on word frequency. By applying the algorithm, the accident case text can be effectively segmented into meaningful units, which serve as the knowledge entities for further analysis and representation in the BOAKG.

Then, keyword extraction. The commonly used methods for feature extraction included word frequency, mutual information, chi-square value, etc. Subsequently, the term frequency and inverse document frequency (TF-IDF) algorithm emerged and presented advantages. The TF-IDF algorithm, initially proposed by Karen Sparck Jones, is widely used in searching and applied to text similarity calculation [33]. The fundamental idea behind TF-IDF is that if a word appears frequently in a document but less frequently in other documents, it carries more significance in distinguishing the document and expressing its core content. Hen2vecce, such words are assigned higher weights [34]. The TF-IDF algorithm calculates a weight for each word in a document based on the term frequency (TF) and inverse document frequency (IDF). Due to its simplicity and effectiveness, the TF-IDF algorithm has become a well-developed and commonly used method in the fields of information retrieval, text mining, and word importance representing. Therefore, the TF-IDF method is utilized for keyword extraction from bridge accident cases. The extracted keywords are considered representative of the accidents. It is calculated using the equation:

$${\mathrm{tf}}_{ij}=\frac{n_{i,j}}{\sum {}_{k}n_{k,j}}$$ (1)

where *tf*_*ij*_ indicates the word frequency of word *i* in document *j*, *n*_*ij*_ is the number of the word in document *j*, and ∑_*k*_*n*_*k*,*j*_ represents the sum of all words in document *j*. *idf* is the frequency of reverse documents and the Equation is

$${idf}_{}=\log\frac{\left|D\right|}{|\left\{j:t\in d_{j}\right\}|+1}$$ (2)

where |D| is the total number of all documents in the corpus, |{*j*:*t*∈*d*_*j*_}| represents the number of documents containing the word *t*_*i*_, and the denominator+1 is to ensure the normal operation when *n*_*i*,*j*_ = 0. The entities could be classified based on the keywords extraction.

The keywords with high frequency are extracted and sorted as nodes. In total, 12 types of knowledge entities are identified and extracted, including accident title, bridge structure, bridge materials, accident position, accident season, accident time period, accident area, accident location (city), level of accident area, accident classification, cause of accident, and accident severity.

The specific description of the accident is used as an attribute, where each attribute value is unique for every case [35]. Attributes of an accident can be extracted to describe the case in multiple dimensions. In this research, six event descriptions are taken as the attributes of the accident, including accident time, specific location of the accident, response measures, accident evolution chain, number of casualties, and economic loss.

#### Extraction of links among knowledge entities

The identification of links is a crucial step in determining the relationships among knowledge entities and constructing the BOAKG. These relationships encompass cause-effect relationships as well as association relationships. Association relationships is the connections between accidents and their consequences, accident reasons, and other related entities. In this research, association relationships are explored specifically between the entity "accident title" and other entities, as outlined in Table 1.

**Table 1:** Links among knowledge entities.

Entity	Link	Entity
accident title	<bridge structure>	bridge structure information
accident title	<bridge materials>	bridge materials information
accident title	<accident position>	accident position information
accident title	<accident season>	accident season information
accident title	<accident time period>	accident time period information
accident title	<accident area>	accident area information
accident title	<accident location (city) >	accident location (city) information
accident title	<level of accident area>	level of accident area information
accident title	<accident classification>	accident classification information
accident title	<cause of accident>	cause of accident information
accident title	<accident severity>	accident severity information

#### Construction of the BOAKG

In the construction of a knowledge graph, knowledge entities and their links are represented by knowledge triples. The construction of the BOAKG involves two types of knowledge triples: <entity, link, entity> and <entity, attribute, attribute value>. All bridge accident cases are stored in the form of triples in a Neo4j database, which is achieved through an automatic Python script. This allows for the construction and presentation of the BOAKG within the Neo4j database environment.

### Accident characteristic attribute weights calculation

Weight calculation methods can be broadly categorized into subjective methods and objective methods. One popular objective method for data weighting is the entropy weight method. This method utilizes entropy, which measures the variability or diversity of an index, to assign weights objectively. However, it may lead to evaluation results that differ from expectations due to a limited number of indicators or inaccurate calculation of indicator data. The Analytic Hierarchy Process (AHP) method is a widely recognized and representative method for subjective analysis weighting. It was introduced and defined by Thomas Saaty in 1977 and has since been utilized as an effective tool for handling complex decision-making problems [36]. The method primarily relies on the subjective analysis and judgment of decision-makers rather than considering actual data of the criteria. It accommodates subjective weighting of factors from the perspectives of different decision-makers [37], and involves directly assigning subjective scores or judgments to determine the weights of criteria or alternatives. Compared to other methods, the calculation process is relatively simple. Besides, AHP is indeed one of the most comprehensive multi-criteria decision-making methods and has found widespread applications in various scientific fields. Furthermore, this approach helps minimize judgmental inconsistencies and requires less information to describe complex decision-making processes [38]. Bridge accidents often involve multiple factors. By applying the AHP method, quantitative assessment and decision-making can be conducted on the accident characteristic attributes. The essential steps of the AHP are as follows:

- Determine the decision goal, criteria, and alternatives in the AHP structure of the decision problem, which serve as the target layer, criterion layer, and alternative layer, respectively.
- Compare the importance of all factors in each criterion layer and create the pairwise comparison matrix.
- Sort each vector at the upper level according to their importance.
- Obtain the total priority value ranking of the scheme level relative to the target level by combining the upper and lower vectors.
- Perform a consistency test on the judgment matrix to ensure logical consistency in the thought process.

#### Construction of the pairwise comparison matrix

Firstly, analyze the relationships among the elements to establish a three-level structure consisting of the target layer, criterion layer, and alternative layer. Then, compare the importance of all elements within each layer and create the pairwise comparison matrix. These comparisons are made by experts who assess the importance of a criterion in relation to other criteria using the AHP value scale. This process is conducted for all elements within each layer, aiming to determine the level of importance and prioritize each sub-factor. Each relevant sub-factor is assigned a relative weight to ascertain its significance. The scaling definition of the matrix can be found in Table 2.

**Table 2:** The scaling definition.

Scale	Meaning
1	Comparing the two factors, the two factors are equally important.
3	Comparing the two factors, one factor is slightly more important than the other.
5	Comparing the two factors, one factor is significantly more important than the other.
7	Comparing the two factors, one factor is strongly more important than the other.
9	Comparing the two factors, one factor is extremely more important than the other.
2,4,6,8	The median of the two neighboring judgments above.

#### Weight calculation

Normalize the elements of the judgment matrix by column according to Eq 3.

$$b_{ij}=\frac{a_{ij}}{\sum_{k=1}^{n}a_{kj}}i,j=1,2,\cdots ,n$$ (3)

where *a*_*ij*_ is the element of the pairwise comparison matrix.

Since the distribution of the weights was approximately reflected by each column in the pairwise comparison matrix, the arithmetic mean of all the column vectors can be used to estimate the weight vector according to Eq 4.


$$w_{i}=\frac{b_{ij}}{n}i,j=1,2,\cdots ,n$$ (4)

Sum the normalized rows and then divided by n to obtain the weight value *w*_*i*_.

#### Consistency test

The purpose of the consistency test is to verify whether the subjective judgments consistent with the quantitative research findings. This test aims to ensure the reasonableness and validity of the research results. When AW = *λ*W, the maximum characteristic root *λ*_max_ can be calculated according to the following Eq 5,

$$\lambda_{\max}=\sum_{i=1}^{n}\frac{(AW)i}{nWi}$$ (5)

The consistency index is calculated through the Eq 6,

$$CI=\frac{\lambda_{\max}-n}{n-1}$$ (6)

### Accident attribute similarity calculation

#### Single attribute similarity calculation

Currently, there are several commonly used classification algorithms, including the Support Vector Machine (SVM) algorithm, Naive Bayesian algorithm, and k-Nearest Neighbor (KNN) algorithm. In the field of Chinese text classification, the SVM algorithm lacks guidance on selecting kernel functions, which makes it challenging to determine the best kernel function for specific classification problems. In the Naive Bayesian algorithm, it tends to yield good results only when there is an extensive number of training samples available. KNN can adapt well to changes in classification standards and offers lower training time complexity compared to the SVM algorithm. Furthermore, it does not make assumptions about the data and is less sensitive to outliers compared to the Naive Bayesian algorithm. Accordingly, this research adopts the KNN algorithm in attribute similarity calculation. KNN is a non-parametric method that was initially developed by Evelyn Fix and Joseph Hodges in 1951, and later improved by Thomas Cover [39]. It has become one of the most popular machine learning algorithms due to its simplicity and accuracy, making it suitable for handling classification and regression problems [40]. KNN operates based on the principle of finding the most similar items or users in a dataset to make predictions. The output value for a test case is determined based on the majority vote or averaging of the output values of its k-nearest neighbors in the training data [41]. By considering multiple neighbors, KNN takes into account the local structure of the data and can provide robust predictions. It can store all the available problems or cases and classify them to the new clusters based on their similarity measure. The outputs are the top k cases with the highest similarity by calculating the similarity between the target case and the source case [42]. The calculation of the single attribute similarity *Sim*(*s*,*t*) between the target case and the source case is based on the following Equations,

Sim(s,t)=1−D(s,t) (7)

$$D(s,t)=\sqrt{\sum_{k=1}^{n}{\left(w_{k}\times D_{k}(s,t)\right)}^{2}}$$ (8)

where *D*(*s*,*t*) is the normalized Euclidean distance between the target case and the source case, *k* is the retrieval attribute number, *n* is the total number of retrieval attributes, *w*_*k*_ is the weight of the attribute *k*, *D*_*k*_(*s*,*t*) is the distance on *k* attribute dimension between the target case and the source case after normalization.

The data types of the retrieved attributes include symbolic type and numerical type, and the 2 data types of *D*_*k*_(*s*,*t*) are determined as follows:

Symbolic type:

$$D_{k}(s,t)=\{\begin{array}{l} 0,P_{sk}=P_{tk}\\ 1,P_{sk}\neq P_{tk} \end{array}$$ (9)

Numerical type:

$$D_{k}(s,t)=1-\frac{d_{k}(s,t)}{{\mathrm{Max}}_{k}{‐\mathrm{Min}}_{k}}$$ (10)

$$D_{k}(s,t)=|P_{sk}-P_{tk}|$$ (11)

Where P_*sk*_ is the k attribute value of the source case and P_*tk*_ is the *k* attribute value of the target case, *d*_*k*_(*s*,*t*) is the distance on *k* attribute dimension between the source case and the target case, *Max*_*k*_ is the maximum value of the *k* attribute value and *Min*_*k*_ is the minimum value of the *k* attribute value in the case base.

#### Comprehensive layer similarity calculation

The comprehensive layer similarity could be calculated based on the Eq 12,

$$\mathrm{Sim}(C)=\sum_{i=1}^{n}W_{i}\mathrm{Sim}(s,t)$$ (12)

Where *W*_*i*_ is the attribute weight, *Sim*(*s*,*t*) is the single attribute similarity.

#### Global similarity calculation

The global attribute similarity could be obtained based on the Eq 13,

SIM(G)=Sim(C)c+Sim(C)g (13)

where *Sim*(*C*)*c* and *Sim*(*C*)*g* is the comprehensive layer similarity of core circle layer and general circle layer.

### The multi-circle layer retrieval strategy

CBR is an intelligent methodology that addresses novel problems by retrieving similar historical cases and adapting their solutions, outcomes, and recommendations [43]. Among the various processes involved in a CBR system, case retrieval holds significant importance [44]. The accuracy of case retrieval greatly impacts the assessment of CBR performance [45]. The BOAKG acts as a database for storing accident information and serves as the case base for case retrieval. Similarity measure methods with multiple formats of attribute values can be found in the practical CBR applications, but the in-depth study is still lacking [46]. In recent years, several extended CBR approaches have been proposed for critical infrastructure risk response [47]. The traditional case retrieval process covers the entire case base and all the characteristic attributes of the cases, in which each attribute presents the same weight during calculation and results in a time-consuming retrieval process. However, it is crucial to recognize that not all case attributes involved in the retrieval hold equal importance. Therefore, this research proposes an innovative multi-circle layer retrieval strategy for bridge operational accident cases. The attributes involved in the retrieval are classified into multiple circle layers based on their importance and level of identification, and different weights are assigned to attributes within each layer.

#### The multi-circle layer characteristic attributes

The 11 accident characteristic attributes used for retrieval are divided into 2 circle layers: the core circle layer attributes and general circle layer attributes. The core circle layer attributes consist of cause of accident, accident classification, and accident severity, which are the attributes with high commonness, high frequency, high differentiation, and high influence. The general circle layer attributes provide additional accident retrieval information and are of secondary importance compared to the core circle layer attributes. The general circle layer attributes include bridge structure, bridge materials, accident position, accident season, accident time period, accident area, accident location (city), and level of accident area. Among these attributes, the attribute “accident severity” in the core circle layer and the attribute “accident time period” in the general circle layer are numerical data, and the other attributes are symbolic data.

In this research, the characteristic attributes are stored and represented using the framework representation method. There are many representing knowledge methods, including frame representation and object-oriented representation, etc. Object-oriented representation is similar to framework representation, but it is more refined in terms of modularity, making it more suitable for the development and implementation of computer languages. However, when it comes to representing and storing characteristic attributes, object-oriented representation may struggle to handle them effectively. Frame representation is one of the most frequently knowledge representation methods mentioned in the literature [48]. The framework representation method focuses on capturing the internal structure of objects. It is rooted in the framework theory and was initially introduced by Minsky, in 1975 [49]. The core idea is to use frames as the fundamental units of expression. Each frame consists of multiple "slots," and each slot can contain a slot value or multiple sides. A side comprises a side name and its corresponding value. The framework representation allows for the analysis of unstructured knowledge and its representation using structured frames. Additionally, lower-level frames can inherit slot values from higher-level frames while also having the ability to supplement and modify them. These advantages make it suitable for handling bridge operational accident cases by covering the informational attributes associated with accident characteristics. Therefore, framework representation method is used in this paper. The two-circle layer characteristic attribute framework is shown in Table 3.

**Table 3:** Characteristic attribute framework.

Slot	Side	Side Value	Type of the side
Core circle layer	cause of accident	overload, collision, fatigue, design defects, water conservancy, quality, others	symbolic data
	accident classification	overload accident, car collision accident, ship collision accident, ice edge collision accident, flood accident, landslide accident, debris flow accident, scouring accident, earthquake accident, landslide accident, design problem accident, water conservancy accident	symbolic data
	accident severity	basically intact(0), slight damage(1), moderate damage(2), serious damage(3), collapse(4)	numerical data
General circle layer	bridge structure	floating bridge, steel frame bridge, arch bridge, beam bridge, suspension bridge, composite system bridge	symbolic data
	bridge materials	wooden bridge, cable bridge, masonry bridge, steel bridge, reinforced concrete bridge, prestressed concrete bridge, hybrid bridge, composite bridge	symbolic data
	accident position	bridge deck, arch, pier, bridge top, bottom, suspender, iron cable	symbolic data
	accident season	spring, summer, autumn, winter	symbolic data
	accident time period	[00:00,5:00)(0); [5:00–6:00)(1); [6:00–8:00)(2); [8:00–11:00)(3); [11:00–13:00)(4); [13:00–17:00)(5); [17:00–18:00)(6); [18:00–24:00)(7)	numerical data
	accident area	northeast region, north China region, east China region, south China region, central China region, northwest region, southwest region	symbolic data
	accident location (city)	city of the accident	symbolic data
	level of accident area	municipalities directly under the central government, provincial capital cities, prefecture level cities, county-level cities, townships	symbolic data

#### The multi-circle retrieval process

In the process of bridge accident case retrieval, the BOAKG is employed as the initial case database to compute the similarity of the core circle layer attributes. Subsequently, select a certain number of cases according to the similarity ranking for the general circle layer selection. Based on the selected cases, the similarity of the general circle layer attributes is calculated. The final global similarity between the target case and the source case is obtained by integrating the two circle layer similarities, and the cases with high global similarity is output as the decision-making cases. The detailed process is described as follows:

a. First round retrieval

Step 1: Input the core circle layer attributes for the first round retrieval, including cause of accident, accident classification, and accident severity.

Step 2: Calculate the weighted comprehensive similarity *Sim*(*k*,*t*) of the core circle layer attributes between the target case and the k-th case in the case base.

Step 3: Sort the obtained *Sim*(*k*,*t*) values in descending order and take the first *n* cases as the core circle type matching results.

b. Second round retrieval

Step 1: Input the general circle layer attributes for the second round retrieval, including bridge structure, bridge materials, accident position, accident season, accident time period, accident area, accident location (city), and level of accident area.

Step 2: Calculate the weighted comprehensive similarity *Sim*(*m*,*t*) of the general circle layer attributes between the target case and the m-th case among the selected n cases. Combining with the core circle layer weighted comprehensive similarity obtained from the first round, the final global similarity result *Sim*(*n*,*t*) could be obtained.

Step 3: Sort the *Sim*(*n*,*t*) values in descending order and obtain the final case retrieval results.

Compared to traditional case retrieval methods, the approach used in this research significantly reduces the number of cases. By retrieving cases based on the core circle layer attributes, the initial set of similar cases is narrowed down. Then, the subsequent retrieval process becomes less complex and the overall retrieval time is reduced.

## Results

### Data collection

#### Topic-specific web crawler

**Fig 1:**
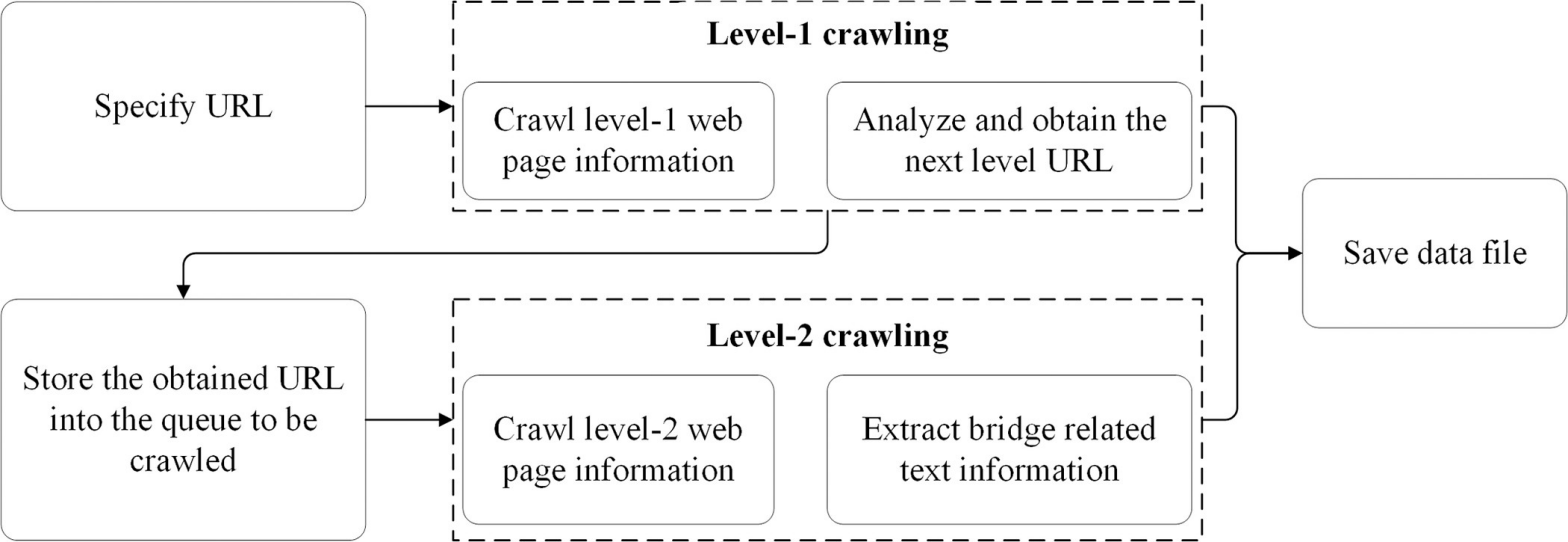
Topic-specific web crawler flow chart.

The research utilized topic-specific web crawler technology to gather relevant web pages related to the interested topics from the internet [50]. This technology enables selective and efficient extraction of specific information. In this research, the topic-specific web crawler technology was employed to obtain the name, time, source, place, and complete contents of the Chinese bridge accident case. The basic flow chart is shown as (Fig 1).

The information in the level-1 web page is the title of the case report, and the information in the level-2 web page is the detailed content of the case report. The name, time, source, place and complete contents of the bridge accident case could be obtained through the crawler process. The name of a case is the title of the news. The time of the case refers to the actual occurrence time. However, in some cases, the report time is not the accurately happening time or the happening time is not clear, which requires manual revision in the later stage. The source of the case is the name of the website, journal, etc. The place of the case is the administrative divisions at the provincial and municipal level in China. The complete contents of the case generally encompass the information about causes, process, results and corresponding measures of the accident. In total, 5462 pieces of original text were collected.

#### Manual screening

Due to the repetition of news reports and irrelevant information crawling, the collected original data need manually screening. Firstly, filtering the accident cases after 1978. Secondly, focusing on the news of the same day or another day according to the time of news release, and integrating the repeated cases. Thirdly, deleting cases with low fitness and relevance. Finally, a total of 324 effective Chinese bridge operational accident cases data were obtained.

### BOAKG modelling

The field of bridge operational accidents is relatively specialized and has clear boundaries, with limited available knowledge. To address this, a down-top approach was employed to establish a BOAKG.

#### Extraction of knowledge entities, attributes, and links

Through the analysis of data with similar faults and causes, the structural data of bridge operational accident can be obtained and the effect of knowledge fusion can be achieved. The Jieba Chinese text segmentation algorithm and the TF-IDF method were utilized for the extraction of knowledge entities. A total of 12 types of knowledge entities were extracted from the collected the collected 324 bridge accident cases, including accident title, bridge structure, bridge materials, accident position, accident season, accident time period, accident area, accident location (city), level of accident area, accident classification, cause of accident, and accident severity. Additionally, 6 attributes of the accident were also extracted, including accident time, specific location of the accident, response measures, accident evolution chain, number of casualties, and economic loss. The association relationships are considered between the entity “accident title” and other entities. In total, 709 entities and 3888 links were extracted, as shown in Table 4. The data is stored in CSV format and imported in Neo4j for further analysis.

**Table 4:** Number of knowledge entities and links.

Category	Knowledge entities/ links	Meaning	Number	Sum
Entities	cAccident	accident title	324	709
	cStructure	bridge structure	6	
	cMaterial	bridge materials	8	
	cPosition	accident position	12	
	cSeason	accident season	4	
	cPeriod	accident time period	8	
	cArea	accident area	7	
	cLocation	accident location (city)	285	
	cLevel	level of accident area	5	
	cTypes	accident classification	11	
	cReason	cause of accident	34	
	cDegree	accident severity	5	
Links	rAccident_Structure	bridge structure in the accident	324	3888
	rAccident_Material	bridge materials in the accident	324	
	rAccident_Part	accident position	324	
	rAccident_Season	accident season	324	
	rAccident_Period	accident time period	324	
	rAccident_Area	accident area	324	
	rAccident_Location	accident location (city)	324	
	rAccident_Level	level of accident area	324	
	rAccident_Types	accident classification	324	
	rAccident_Reason	cause of accident	324	
	rAccident_Degree	accident severity	324	
	rAccident_Time	Accident occurrence time	324	

#### BOAKG modelling construction

**Fig 2:**
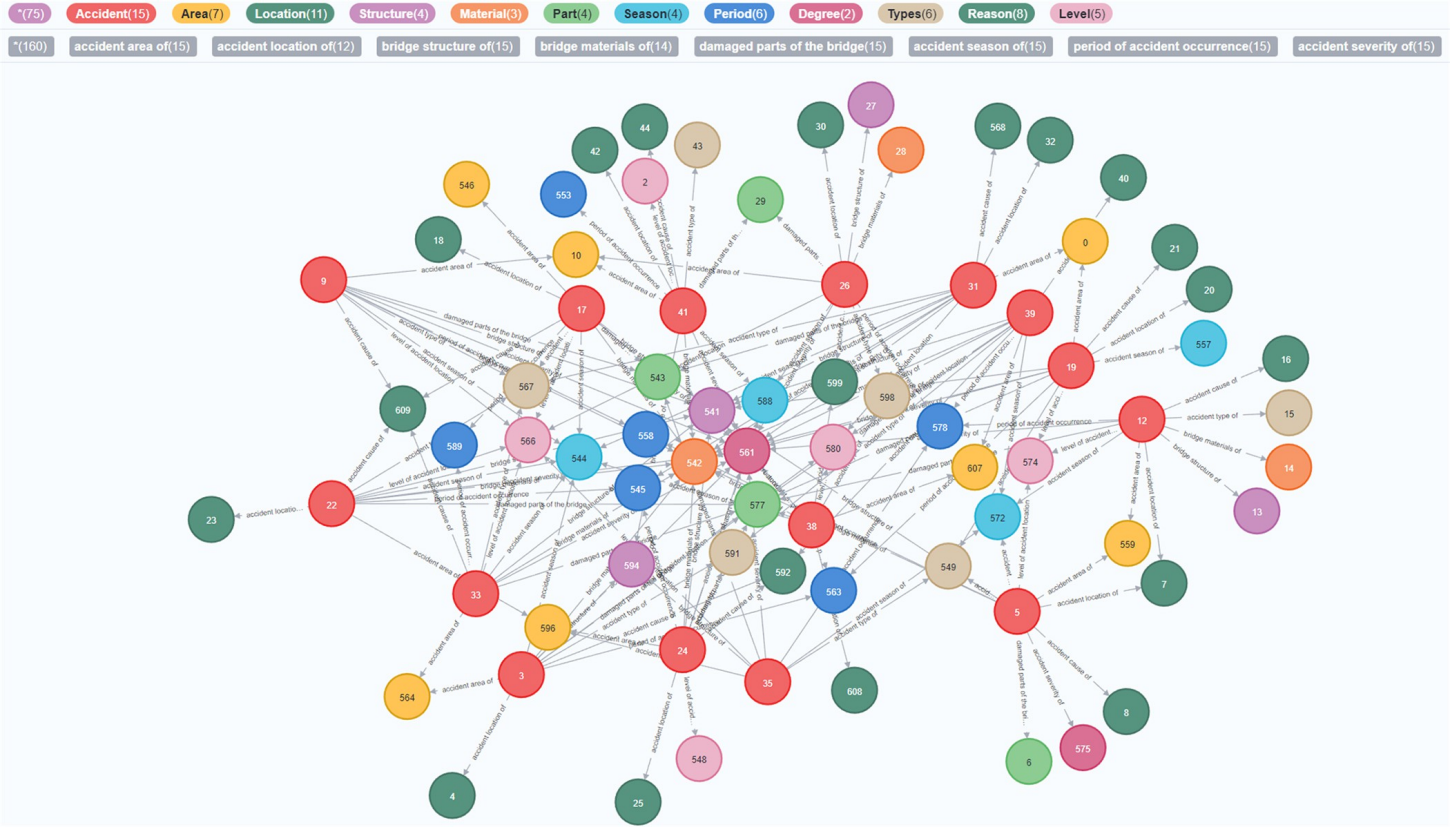
The BOAKG.

Based on the knowledge extraction process, the bridge accidents can be represented in the form of two types of knowledge triples: <entity, link, entity> and <entity, attribute, attribute value>. All the bridge accident cases are stored in the Neo4j database in the form of triples through automatic build of Python script. The constructed BOAKG modelling is shown as (Fig 2).

### Case retrieval validation

#### Structure the decision hierarchy of bridge operational accident

**Fig 3:**
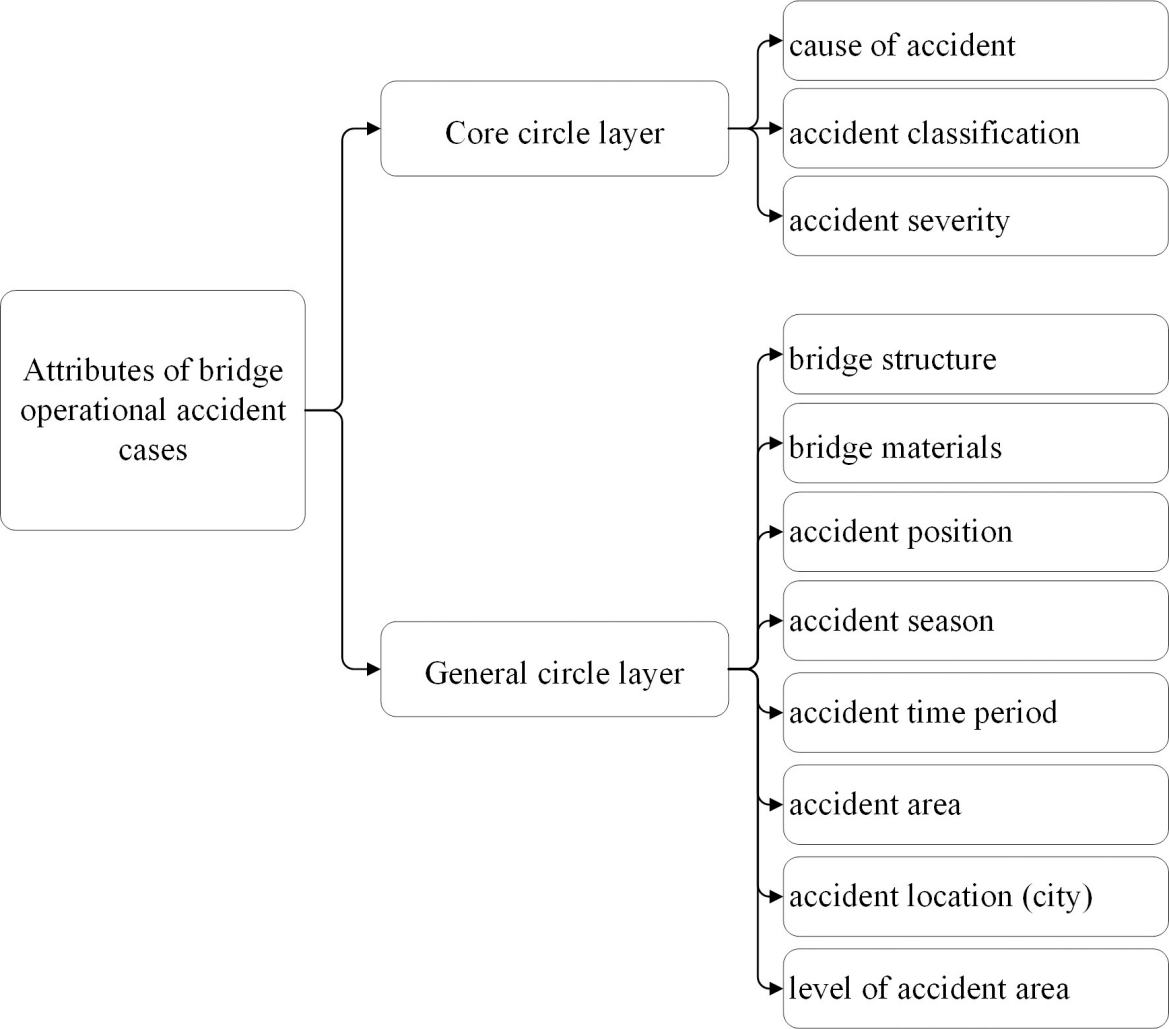
The decision hierarchy of bridge operational accident.

Structure the decision hierarchy of bridge operational accident with a top-down approach based on the multi-circle layer retrieval strategy. The hierarch consists of the target layer at the top, the intermediate criterion layer, and the lowest alternative layer, as shown in (Fig 3).

#### Pairwise comparison matrix construction and weight calculation

Five experts were invited to discuss the pairwise comparison of the elements in each layer. All the members of expert panel have earned their doctorate degrees in engineering management. They also have more than ten years of working experiences in bridge operation management or research. AHP approach was applied after the hierarchy developed. Three pairwise comparison matrices were obtained through pair-wise comparison in the three layers based on the five experts’ comprehensive judgement opinions. Specifically, each expert provided independent scores, resulting in three pairwise comparison matrices, respectively. Further, the average score from the five experts were computed and three integrated pairwise comparison matrices were constructed. Finally, the weights were calculated according to the Eq 3 and Eq 4, as shown in Table 5.

**Table 5:** The weights calculation results.

Criteria	Criterion layers and Alternatives	W_i_
The two layers	The core circle layer	0.9000
	The general circle layer	0.1000
The core circle layer	Cause of accident	0.4440
	Accident classification	0.1762
	Accident severity	0.2797
The general circle layer	Bridge structure	0.0103
	Bridge materials	0.0099
	Accident position	0.0151
	Accident season	0.0178
	Accident time period	0.0218
	Accident area	0.0113
	Accident location (city)	0.0059
	Level of accident area	0.0080

#### Consistency test

Based on the pairwise comparison matrices, *λ_max_* can be respectively calculated according to Eq 5, which are 2.0000, 3.0536, and 8.4807. The consistency test is conducted according to Eq 6 and the results are presented in Table 6. According to the results, the values calculated by the three pairwise comparison matrices are consistent with CI<0.1, which satisfy the consistency test standard.

**Table 6:** The consistency ratio of the judgment matrix.

The judgment matrix	The circle layer	The core circle layer	The general circle layer
CI	0.0000	0.0516	0.0487

#### Similarity calculation

Three target cases were selected for the case retrieval algorithm validation. Two parameters, n and k, were set for each test case. Here, n represents the top n cases selected for the first round retrieval, and k represents the top k cases selected for the second round retrieval. The number of nodes that successfully matched all cases in the case base was counted and the feasibility of the retrieval model was validated. The selected three target cases are “7.7 Collapse Accident of Qili Bridge in Liu’an City, Anhui Province”, “5.12 Collapse Accident of Shoujiang Bridge in Wenchuan County, Sichuan Province”, and “6.23 Ship Collision Accident of Jinshan Hengxi East Street Bridge in Shanghai”, which are abbreviated as “7.7”, “5.12”, and “6.23”, respectively. The attribute information of the three target cases is shown in Table 7.

**Table 7:** The attribute information of the three cases.

Accident/Attribute	7.7	5.12	6.23	Circle layer
Cause of accident	water erosion	earthquake	ship collision	core circle layer
Accident classification	water conservancy accident	earthquake accident	ship collision accident	
Accident severity	collapsing	moderate damage	slight damage	
Bridge structure	beam bridge	beam bridge	beam bridge	General circle layer
Bridge materials	reinforced concrete bridge	reinforced concrete bridge	reinforced concrete bridge	
Accident position	bridge deck	bridge deck	bridge pier	
Accident season	summer	spring	spring	
Accident time period	forenoon	afternoon	noon	
Accident area	east China region	southwest region	east China region	
Accident location (city)	Liu’an City, Anhui Province	Wenchuan County, Sichuan Province	Shanghai	
Level of accident area	prefecture-level City	county-level cities	municipalities directly under the central government	

In the case base, there are a total of 324 cases numbered from 1 to 324. The similarity calculation includes evaluating attribute similarity of the attributes similarity in the core circle layer, the general circle layer, and the global similarity. For the 3 cases, the values of the parameters n and k are set as 15 and 5, respectively. Taking the first case “7.7” as an example to show the retrieval results, as presented in Table 8.

**Table 8:** The retrieval results of the case “7.7”.

The core circle layer		The general circle layer		The global similarity
The retrieval case	The similarity	The retrieval case	The similarity	
64	0.9000	240	0.1	1.0000
256	0.9000	228	0.0829	0.9829
116	0.9000	193	0.0747	0.9747
114	0.9000	244	0.0747	0.9747
113	0.9000	64	0.0709	0.9709
147	0.9000			
30	0.9000			
244	0.9000			
310	0.9000			
228	0.9000			
46	0.9000			
193	0.9000			
47	0.9000			
179	0.9000			
321	0.9000			

The retrieval results for the 3 target cases are shown in Table 9.

**Table 9:** The retrieval results for the 3 target cases.

Target case	Top 5 matching cases	The global similarity
240 (7.7)	240	1.0000
	228	0.9829
	193	0.9747
	244	0.9747
	64	0.9709
175 (5.12)	175	1.0000
	174	0.8462
	42	0.8392
	45	0.8370
	43	0.8370
103 (6.23)	103	1.0000
	35	0.9829
	76	0.9652
	77	0.9621
	311	0.9565

According to the retrieval results, the top 5 cases exhibit a high similarity in the core circle layer, with all values above 0.8000. However, only several cases show high similarity in general circle layer. The retrieval results indicate the significance of the core circle layer attributes. It is worth noting that the global similarity of the top 5 cases is also above 0.8000, affirming the feasibility of the case retrieval algorithm.

## Discussion

Based on Table 9, it is observed that each target case has a similarity score of 100% when matched with itself. Furthermore, the table suggests that the target cases can also be matched with other cases that share the same type of accident or cause. These matching cases exhibit a relatively high degree of similarity to the target case. For example, "7.7 Collapse Accident of Qili Bridge in Liu’an City, Anhui Province" has similar cases such as "No.228-8.14 Collapse Accident of Shipuhead Bridge in Lishui, Zhejiang Province" " No.193-7.1 Collapse Accident of Jiulongwan 2# in Xinhua Line, Lvliang, Shanxi Province," and "No.244-7.11 Collapse Accident of Chuanxi Bridge in Chengdu, Sichuan Province", all of which are hydraulic engineering accidents. Similarly, accidents "5.12 Collapse Accident of Shoujiang Bridge in Wenchuan County, Sichuan Province" have similar cases like " No.174 case" "No.42 case" and "No.45 case", all of which are bridge accidents caused by earthquakes. Finally, the "6.23 Ship Collision Accident of Jinshan Hengxi East Street Bridge in Shanghai" has similar cases such as " No.35 case" " No.76 case" and " No.77 case", all of which are bridge accidents caused by vessel collisions. These similar cases have relatively high match scores, and the differences in overall similarity usually come from the differences in some individual general-level nodes. By using the similarity matching verification process in this study, it can effectively find the most similar cases to the target case, which provides powerful support for subsequent case-assisted decision-making. These results demonstrate that the proposed case-matching method is highly reliable and effective.

Furthermore, in the "3.3 Case Retrieval Validation" section, the multi-circle layer retrieval strategy (with n = 10 and k = 5) successfully matches a total of 1052 nodes based on the number of matching nodes. However, the nearest neighbor retrieval algorithm would require matching a total of 3564 nodes. Compared to the nearest neighbor matching algorithm, the retrieval strategy proposed in this paper can eliminate a large number of irrelevant cases in the first matching, resulting in a significantly lower total number of matched nodes. In contrast, traditional nearest neighbor algorithms require traversing all case nodes, resulting in lower matching efficiency. Therefore, the method proposed in this paper can improve the efficiency and accuracy of case matching.

However, the research in this paper still has some limitations. In the field of bridge accident cases, the application of knowledge graph construction technology is still in its early stages. Although the method proposed in this paper establishes a knowledge graph-based bridge accident case base, further collection of more case samples is needed to enrich the case repository. It should be noted that this research has only collected 324 cases, which limits the utilization of the current BOAKG. The scope of this study is limited to accidents that occur during the operation of bridges in China. Future research can expand the scope of study to compare bridge accident cases and response measures in different countries or regions. To achieve this goal, more efficient case representation methods and multi-source information fusion techniques need to be considered. The cases should be collected as many as possible, including detailed information on accident precursors, causes, processes, outcomes, and response measures. Besides, the establishment of a cross-lingual, cross-cultural bridge accident data standard and fusion approach should be adopted. In this study, the BOAKG is designed as an open network capable of continuously accumulating new cases over time. This means that as more bridge operational accident cases are added to the knowledge graph, its utility and effectiveness in case retrieval will increase, further enhancing the knowledge base for future decision-making and response management.

## Conclusions

Case representation and similar case retrieval are the key contents of understanding bridge operational accidents and conducting automated reasoning. In terms of case representation, a knowledge graph has been adopted to facilitate the multi-dimensional network modeling of bridge operational accidents. This approach addresses the current situation where these accidents are primarily stored in unstructured documents, resulting in low efficiency and effectiveness in information retrieval. The established BOAKG organizes the knowledge related to bridge operational accidents in a structured and orderly manner, drawing upon principles from knowledge engineering. Its purpose is to process and store knowledge in a more systematic way. It enables automated and rapid information retrieval for cases, as well as facilitating the visualization display of case information. The BOAKG serves as a database for subsequent case retrieval tasks. CBR is a valuable method for analyzing bridge operational accidents. An innovative multi-circle layer retrieval strategy based on CBR has been proposed for case retrieval. The multi-circle layer retrieval strategy proposed in this study demonstrates high retrieval efficiency and feasibility for case retrieval. The results of the retrieval demonstrate that the global similarity can exceed 0.8000, indicating the feasibility of the case retrieval algorithm and an acceptable retrieval effect. Besides, by increasing the number and diversity of cases in the database, the retrieval results can be subsequently optimized.

The study expands the application scope of knowledge graph and CBR from a theoretical perspective and explores the mechanism of combining these two methods to achieve intelligent decision-making for infrastructure operational risks. In practical terms, this research provides a valuable methodology for bridge operation management departments, particularly in the context of risk intelligent management. Additionally, it can also serve as a reference for risk management in other infrastructure operations. The innovation of this study is twofold. Firstly, it lies in the research content, which focuses on bridge operational risk by analyzing cases of bridge operational accidents. This approach contributes to a deeper understanding of bridge operational risks. Secondly, the study innovates in terms of research methodology by utilizing knowledge graph technology from the field of artificial intelligence and integrating it with CBR technology. Notably, the paper introduces a novel multi-layer retrieval strategy in the application of CBR, which enhance the efficiency and effectiveness of case retrieval.
